# Women’s reasons for, and experiences of, choosing a homebirth following a caesarean section

**DOI:** 10.1186/s12884-015-0639-4

**Published:** 2015-09-03

**Authors:** Hazel Keedle, Virginia Schmied, Elaine Burns, Hannah G Dahlen

**Affiliations:** School of Nursing and Midwifery, Western Sydney University, Locked Bag 1797, Penrith, NSW 2751 Australia

## Abstract

**Background:**

Caesarean section is rising in the developed world and vaginal birth after caesarean (VBAC) is declining. There are increased reports of women seeking a homebirth following a caesarean section (HBAC) in Australia but little is known about the reasons for this study aimed to explore women's reasons for and experiences of choosing a HBAC.

**Methods:**

Twelve women participated in a semi-structured one-to-one interview. The interviews were digitally recorded, then transcribed verbatim. These data were analysed using thematic analysis.

**Results:**

The overarching theme that emerged was ‘It’s never happening again’. Women clearly articulated why it [caesarean section] was never happening again under the following sub themes: ‘treated like a piece of meat’, ‘I was traumatised by it for years’, ‘you can smell the fear in the room’, ‘re-traumatised by the system’. They also described how it [caesarean section] was never happening again under the sub themes: ‘getting informed and gaining confidence’, ‘avoiding judgment through selective telling’, ‘preparing for birth’, ‘gathering support’ and ‘all about safety but I came first’. The women then identified the impact of their HBAC under the subthemes ‘I felt like superwoman’ and ‘there is just no comparison’.

**Conclusions:**

Birth intervention may cause physical and emotional trauma that can have a significant impact on some women. Inflexible hospital systems and inflexible attitudes around policy and care led some women to seek other options. Women report that achieving a HBAC has benefits for the relationship with their baby. VBAC policies and practices in hospitals need to be flexible to enable women to negotiate the care that they wish to have.

## Background

The caesarean section rate has increased in Australia from 27 % in 2002 to 32.3 % in 2011 [1] with a large number of women potentially considering a VBAC for a second or subsequent baby. VBAC is defined as “…a vaginal delivery [birth] by a woman who has had a previous caesarean delivery.”([2], p.4, [3]). Australian national data indicates the rate of Vaginal Birth after Caesarean (VBAC) is 12.3 % [1] with success rates varying between 14 and 90 % [4–6]. The rate is highest in the public sector and lowest in the private sector.

The risks involved in repeat operative deliveries for subsequent pregnancies include an increase in operative trauma, placenta praevia and accretia, surgical injury and hysterectomy [7]. There are many benefits for women, their families and society when women are supported to have a VBAC. Vaginal birth leads to quicker post-birth recovery, less operative trauma and rates of endometritis, shorter lengths of hospital stay, and improved feelings of wellness for women [8–11]. For birthing women the benefits can also include the experience of pride and achievement which can lead to a period of psychological healing following previous birth trauma [12]. Birth trauma can include the way a woman was treated by health professionals during her pregnancy and labour and birth care, as well as through the mode of birth she experienced. The definition of birth trauma is individualised, and as Beck states, ‘ is in the eye of the beholder’ ([13], p.304).

While most health providers’ recognise VBAC as a valid birth choice, it is often discouraged in both overt and covert ways. Health providers express concerns about safety, backup for emergencies and medico-legal risk [14, 15]. If women do have a VBAC, most typically they choose to birth in hospital, but there are some women who choose to have a VBAC at home [4]. In this paper the term HBAC is also used. The latest Australian statistics indicate that 0.4 % of births are planned home births, although it is argued that some may go unreported [1, 16].

The majority of research surrounding VBAC focuses on the rates of uterine rupture and factors that may affect this rate [17–20]. VBAC undertaken at home is discussed in the literature but there are few studies that have specifically looked at the safety of VBAC at home. A recent German study found that women planning an out-of-hospital VBAC (birth centre and homebirth) had a 77.8 % success rate compared to in-hospital VBAC of 32 % in the same region [21]. The study also concluded that out of hospital VBAC was safe where there were appropriately qualified midwives and clear risk screening criteria [21]. The higher VBAC rates are also found in studies that look at the safety and outcomes of births undertaken at home or in birth centres compared with hospital births, ranging from 73.5 to 96 %, the average being 87.6 % [4, 22–24]. In comparison, Australian statistics indicate that only 12.3 % of women with a history of previous caesarean had a normal vaginal birth [1].

This paper aims to explore the reasons for, and the experiences of, women who choose to have a HBAC. This group of women can offer invaluable insights into the decision-making and experiences of women when choosing to have a VBAC at home. These insights may in turn help health professionals understand the factors that are important to women planning a subsequent birth after caesarean section.

## Methods

A qualitative interpretive approach underpinned by a feminist framework informed this study. Qualitative research was chosen as it allows for the ‘understanding of a particular phenomenon from the perspective of those experiencing it’ ([25], p.398).

A feminist framework is grounded in the knowledge that contemporary western society is patriarchal and hierarchal [26]. In particular, in relation to this study, a feminist approach provides an appropriate lens to consider the gender and power relationships that can occur within the health care setting [27]. As described above, the dominant biomedical health system identifies VBAC and homebirth as risky. With this in mind, a feminist approach provides an appropriate theoretical framework when exploring why women choose to go against the dominant cultural beliefs or practices and have a VBAC at home. Qualitative interview techniques facilitate exploration of women’s experiences and enable the formulation of deeper knowledge about the factors influencing the choices women make [26].

As a researcher I had ‘insider’ status with the selected cohort for this study as I had planned a HBAC that resulted in a hospital VBAC. I am also a privately practising homebirth midwife who supports women who plan to have a HBAC. This information was shared with participants and allowed for an easy rapport at the beginning of the interviews. Researchers who have had previous experience, relevant to the group being researched, have reported benefits from this insider status such as gaining rapport and accessing the group [28].

Reflexivity was particularly important in this study as I held the status of insider. Reflexivity can be seen as ‘the process through which a researcher recognises, examines, and understands how his or her own social background and assumptions can intervene in the research process’ ([29], p.21). Reflexivity increases validity and transparency in research [30] and is necessary through all stages of the research process [31].

I utilised reflexivity in order to ensure that my background and affinity with participants did not influence the data gathered by setting myself in the ‘middle ground’ of the role of the researcher and held the belief that shared experiences can be interpreted differently for each individual [28]. To ensure this I kept field notes and memos about my experience, my thoughts and feelings as I interviewed the women and undertook the analysis. I made a point of noting how my own views or experiences were consistent with what women had stated in an interview as well as identifying the times their perspective differed. This can be seen in the following excerpt from field notes:

I found that I was challenged today when one of the women said her partner felt more involved in the hospital VBAC than in the HBAC as I thought that would be the other way round. She thought it was due to having a better support team rather than just him (field notes).

Ethics Approval was gained through the University of Western Sydney’s Human Research Ethics Committee (approval number H9853). Women have been de-identified and given pseudonyms.

### Participants and recruitment

A range of recruitment strategies were used to gain access to potential participants such as advertising on home birth specific webpages, social network sites and through informal snowballing techniques. Social networking was the most successful recruitment strategy with 36 women making contact within 48 h of a social networking advertisement. Women were invited to make contact via email and information on the study and consent forms were sent by return email.

The inclusion criterion was women who had achieved a VBAC at home within the last 5 years. The number of previous caesareans or previous vaginal births was not identified within the inclusion criteria and as such women with more than one caesarean or vaginal birth were included. Four women were excluded due to not fulfilling the inclusion criteria for the time frame or were planning to HBAC with a current pregnancy. These women were contacted by phone and thanked for their willingness to be involved and given an explanation on why they were unable to be part of the study, the women were understanding and expressed their excitement for the study. The time frame of within the last 5 years was included to allow for recency of experience within the current climate of homebirth in Australia. Sixteen women did not return consent forms and were unable to move on to the next level of the study and two women were unable to commit to interview. The lack of returned forms may be related to the necessity to print, sign and either scan and email or post back to the research team that may have been restrictive without the necessary devices or time. In total twelve women across Australia returned consent forms and could be interviewed.

### Data collection

Interviews were used to collect data. Eight women from my home State of NSW participated in face-to-face interviews held in their own home. Telephone and Skype was used for the women in States other than NSW which included Western Australia, South Australia, Victoria and Queensland. Demographic information was collected from all participants.

**Fig. 1:**
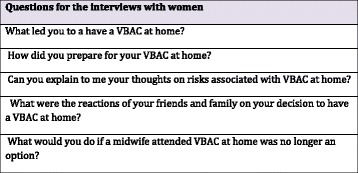
Interview questions for women

Interview questions are presented in Fig. 1. These questions were used during all the interviews as well as specific questioning used for clarification of information. On average the interviews were 56 min for the face-to-face interviews and 34 min for the telephone interviews. Reciprocity was an important component of the interview and interaction with the participants. Reciprocity, is an important element of feminist research and involves the mutual sharing of information to create an open environment for the interview [26]. Building a rapport between researcher and interviewee facilitates the breakdown of the traditional hierarchal relationship that can occur with the resulting benefit of a relaxed interview and an in depth sharing of VBAC stories and this was achieved by identifying shared experiences between the interviewer and interviewee [32].

### Analysis

Thematic analysis was used to analyse the data set [33].

‘Thematic analysis involves the searching across a data set – be that a number of interviews or focus groups, or a range of texts – to find repeated patterns of meaning’ ([34], p15).

The process commences with the transcribing of interviews and then reading and re-reading over the transcripts. The fluid nature of thematic analysis occurs with coding of data items, developing lists of codes, identifying themes across the data set then returning to the data items and deciding on their relevance and fit to the themes. Themes develop out of the codes and the research story determines the overarching theme from the themes and subthemes [25, 34].

The data was managed using the qualitative coding software NVIVO.

As a Masters Honours student I undertook the preliminary analysis, and the three other authors reviewed some of the transcribed data and discussed and checked the interpretations made during data analysis.

Trustworthiness is made up of credibility, transferability, dependability and conformability [35, 36]. In this study this was demonstrated in the use of reflexivity, describing the audit trail and field notes, the involvement of my supervisors during data collection and analysis and the identification of similar themes in similar studies.

## Results

### Participant characteristics

Participants ranged from 26–40 + yrs with the majority between 31 and 35 years and they came from metropolitan areas across Australia. Participants predominantly had tertiary level education, with one woman completing her high school education and the remaining women identifying as having qualifications at certificate/diploma level, degree and postgraduate levels. Two women had more than one previous caesarean, one woman having four previous caesareans and one woman having two previous caesareans. Four women had a VBAC in hospital prior to their HBAC. The majority of the women (10/12) employed a privately practicing midwife (PPM) for their HBAC but one woman had an unregistered birth worker and another woman had a freebirth. The participant demographics are presented in Table 1.

**Table 1:** Demographic details of women

Women	Age	Marital status	Employment	Antenatal model of care	Previous births	Qualifications	State
1	39	Married	Part time	^a^PPM	3XNVB	Certificate/Diploma	NSW
					4XC/S		
					1XVBAC		
					1XHBAC		
2	30	Married	Part time	PPM	2XC/S	University Degree	NSW
					1XHBAC		
3	33	Married	At home	PPM	1XC/S	University Degree	NSW
					1XHBAC		
4	35	Partner	Part time	PPM	1XC/S	Postgraduate Degree	NSW
					1XHBAC		
5	40	Married	Full time	PPM	1XC/S	Certificate/ Diploma	NSW
					1XVBAC		
					1XHBAC		
6	35	Married	Not identified	PPM	1XC/S	Certificate/ Diploma	NSW
					1XVBAC		
					1XHBAC		
7	38	Partner	At home	PPM	1XC/S	High School Certificate	NSW
					1XHBAC		
8	34	Married	At home	PPM	1XC/S	Certificate / Diploma	NSW
					1xHBAC		
9	33	Married	At home	PPM	1XC/S	Certificate / Diploma	VIC
				Midwives clinic	1xHBAC		
10	34	Married	At home	Unregistered birth worker	1XC/S	Certificate / Diploma	SA
					1xHBAC		
11	29	Partner	Self employed	Freebirth	1XC/S	Certificate / Diploma	QLD
					1XVBAC		
					1XHBAC		
12	30	Married	At home	PPM	1XC/S	University Degree	WA
					1xHBAC		

^a^PPM Privately Practising Midwife

### Overarching theme

The overarching theme that emerged from the data was “It’s [caesarean section] never happening again”. This overarching theme captured the strong intent of the women that gave birth vaginally following a previous, often traumatic, birth or births. For many of the twelve women interviewed the decision to have a HBAC followed a previous traumatic birth experience as Anne described:

As I started to heal, as I started to feel less guilty about it and less like I’d done something wrong, I started to get angry and that’s what made me determined and I said, you know, “It’s never happening again, that’s it.”

It’s happened to us twice now…next time we’re going to do it right (Anne).

The overarching theme ‘it’s never happening again’ comprised three themes, ‘why it’s never happening again’ and ‘how it’s never happening again’ and ‘the impact of HBAC’. Each theme had related subthemes that can be found in Table 2.

**Table 2:** Themes and subthemes

It’s never happening again	
Theme	Sub Theme
Why it’s never happening again	Treated like a piece of meat
	I was traumatised by it for years
	You can smell the fear in the room
	Re-traumatised by the system
How it’s never happening again	Getting informed and gaining confidence
	Avoiding judgment through selective telling
	Preparing for birth
	Gathering support
	“All about safety, but I came first”
The impact of HBAC	“I felt like superwoman”
	There is just no comparison

In ‘why it’s never happening again’ the women describe how they were determined not to repeat the past experience of birth by caesarean section through reflecting on their previous birth experience and the strong feelings that motivated them to make sure their previous birth trauma is not repeated. In ‘how it’s never happening again’ women discussed how they sought out other options and then prepared for and ultimately achieved their goal of a vaginal birth after caesarean. ‘The impact of HBAC’ describes the joy and feelings of achievement associated with a successful HBAC and also compared the different models of care they had experienced.

Within each of the three themes there were subthemes that developed and within these subthemes there were a number of key concepts. For the purpose of this paper the sub themes will be discussed with reference to some of the key concepts.

### Why it’s never happening again

This first theme describes the women’s experiences of their caesarean experiences and in some cases their attempt to VBAC in hospital. The strong feelings that these experiences created often resurfaced when the women approached health care providers to assist them with a VBAC. The subthemes that occur in this theme are ‘treated like a piece of meat’, ‘I was traumatised by it for years’, ‘you can smell the fear in the room’ and ‘re-traumatised by the system’.

### Treated like a piece of meat

Although many of the women in the study had experienced a vaginal birth the interviewees commonly commenced with a reflection of their CS experience. The women were able to recall in detail the experience of having had a caesarean section. They remembered how the health professionals acted and what they had said during the operation and the casual and impersonal nature of this had lasting effects on them.

God even now it brings up emotions [silence, quiet crying] … just I mean the experience of having a caesarean so you, you are cut open and then they take the baby and I’m deliriously happy that he’s, that he’s been born [crying], sorry… but the doctors, but their talking about their weekend while they are stitching me up, and they are talking about what they are going to do at the weekend, you just feel like a piece of cold meat on the slab, and I remember saying excuse me and asking them a question and it was like it was a big thing (Jeanne).

Women described feeling loss of control throughout the pregnancy and birth continuum, not just at the caesarean. Being treated like a piece of meat meant that women often felt ignored in the process and not involved in the decision making process. Examples of this include being given medications without informed consent or refusal to provide adequate birthing props such as birthing mats to help the woman gain comfort.

I ended up caving, even though I wanted a natural birth…I couldn’t get comfortable. Even though I’d realised I was having a back labour, no one could get me a mat for my knees, the bung [cannula] was really hurting my hand, I couldn’t get down on all fours…. I ended up asking for an epidural…I was told to sit back, have a cup of tea. I kept wanting certain things, wanting to lean forward but I just got told to sit back in the bed. After a period of time my obstetrician phoned and said that I’d better get my head around the fact that baby wasn’t going to be born vaginally, so all that was done via phone call… and that I should suddenly consent and have a caesarean, and so I did (Natalie).

### I was traumatised by it for years

The women often identified that the stress and trauma from the birth experience did not become evident for several weeks, even months after the birth and often remained with them for ‘years’. Women described the process of identifying and working through the trauma surrounding the initial caesarean experience. For some women this also included subsequent caesareans.

At about 4 weeks my primary midwife was talking to me about how I wasn’t really sleeping, and she… helped me realise that it was me that was awake and not the baby, and that I was actually like replaying this surgery…that was what I was recalling, not the birth, but surgery, over and over in my mind and it made me sort of realise that I needed to speak to someone about it, (Mary).

Women also described feelings of regret, self-blame, isolation and feeling robbed.

It was really out of the blue, and I really did grieve for…the loss of my natural delivery, I had been so looking forward to it, and I had always said up to that point, “If I end up having a caesarean I will feel really robbed. And I did, and for a couple of weeks after my birth…I would just cry, spontaneously, just cry, because I would be so upset about it (Carol).

### You can smell the fear in the room

For the five women in the study who had previously experienced a VBAC (or attempted a VBAC) in the hospital system prior to their HBAC there was a period of reflection in the interviews where they discussed these experiences.

We went to the hospital, so contractions were coming every minute or so and then I get there and its freezing cold and instantly its uncomfortable …I’ve got a drip in cos I’m group b strep positive …and I don’t know, you can smell the fear in the room (Jeanne).

Although the environment of the hospital was identified as having a negative impact in all cases, the attitude and behaviours of the health professionals in the hospital environment had a greater negative impact on their experiences. Women found that health professionals often appeared uncomfortable to care for women who wished to achieve a VBAC.

My obstetrician … agreed to no monitoring for the first bit but then all of a sudden I was strapped down in the stirrups with the monitor on in the end, it was quite horrible (Amy).

When women were reflecting on the interactions they had with health professionals in previous births or in the preparation for the VBAC, obstetricians played a key role. Women expressed surprise at the negative attitudes directed at them around natural birth and VBAC.

“But I just don’t understand why you’d want to have a vaginal birth.”[Obstetrician] And I said, “Well, it’s not very nice to have your stomach cut open and a baby ripped out of it.” And he [Obstetrician] said, “Well I can’t imagine it’s very nice to push it out of your vagina either.” And [laughs] and I was just…I, I…my jaw hit the floor, because I thought you’re an obstetrician! You’re…this is your job, and you’re telling me…like, I just couldn’t understand it. Because I find obstetricians don’t, they don’t believe in birth. They’re…they’re surgically trained, you know? Not naturally trained (Anne).

This sub theme reflected the experiences of women who attempted (and in some cases achieved) a VBAC in the system. The obstetricians mostly did not support women wishing to have a VBAC and expected the women to elect for a repeat caesarean.

### Re-traumatised by the system

The majority of women stated that at the start of the HBAC pregnancy they had approached a hospital to discuss their care and desire to avoid a range of interventions and to experience a VBAC. They were concerned that the cascade of intervention would happen again. In the interview they reflected on the unsupportive and negative attitudes directed at them. Many of the women approached the VBAC in hospital with the attitude that this time they would negotiate the interventions that they would agree to and those that they wished to avoid.

When attempting to negotiate the VBAC women reported that they were subject to varying degrees of intimidation and bullying by hospital staff, from repeated lecturing to disrespectful scaremongering. Some women found that at each antenatal appointment they were told about the same restrictions that would occur during labour, such as continuous monitoring, IV therapy and no water births and there was a lack of willingness on behalf of health professionals to compromise on any of these.

I have never tried harder in my life to compromise, and that was the thing that just blows my mind … I was …trying to make the system actually fit this compromise that isn’t there, And even to the point where I said, “I’m actually saying…like, I put it in my birth plan that we’re going to say no to continuous monitoring and things like that.” And they were just saying, “Well, you can’t”. (Laura).

The health professional would use language such as ‘catastrophic’ and ‘dead baby’ to coerce the women into agreeing to their policies.

When I was pregnant with Jo I was told by a doctor that I was most likely going to die and that my son Jack wouldn’t have a mother to go home to and to embark on a VBAC was stupidity so, I knew that was ridiculous but, and it still affects you (Jeanne).

Some of the women articulated how the intimidation and lack of co-operation they experienced in planning their VBAC in hospital reinforced the residual feelings they had from their traumatic birth. For some women this was the motivation to become educated about VBAC and to find an alternative way to achieve a VBAC. This ultimately put the women onto the path of choosing a HBAC.

### How it’s never happening again

Once the barriers of VBAC in the hospital had been recognised the women in this study described the journey they undertook in becoming educated and confident in their ability to have a VBAC. The women identified that the unfolding of their decision to HBAC was weaved throughout the gaining of knowledge, the gathering of support and in the majority of women, hiring a privately practising midwife (PPM). This second theme describes the methods, resources and support that the women utilised during their journey of achieving a HBAC.

It seemed to be that the only way we were going to be able to do it was to come home. Simple as that (Anne).

### Getting informed and gaining confidence

The women in this study took steps to ensure that they were knowledgeable about the factors that increase the risk for uterine rupture. Some women identified that the risk of birthing in hospital included being pressured to have a repeat caesarean or being subject to the cascade of intervention, including early induction.

I think the risks are higher in a hospital as there are less chance of the VBAC being successful, more chance you’ll be exposed to intervention you don’t need and at the end of the day all of that intervention is a higher risk than having a straight VBAC with nothing else involved (Jodie).

Women in this study explored not only the risk of uterine rupture but also the risks of repeat caesarean, on their body, and their baby. This information came from health professionals, personal contacts and from research.

I think the decisions to research and understand risk and act in what you see as your best interest in understanding them… so that taking that control, understanding the risks, making those decisions I think is really important and I think it is a shame that it doesn’t happen more (Susan).

### Avoiding judgment through selective telling

Once the decision to have a homebirth was made women faced the hurdle of telling family and friends about their choice. Homebirth is not a mainstream option in Australia so women anticipated the reactions could potentially be negative and damaging. Some women found that avoiding telling people was a preferred option, some avoided associating with those whom they knew would be against home birth and others chose to wait until the baby was born before disclosing their HBAC choice.

When we announced the birth, we sent everyone a text message that said you know, was born via a planned homebirth into water at home …and we dealt with it then … if it didn’t work out, I didn’t want to have to tell everyone why it didn’t work out (Louise).

The women did not expect positive reactions from the hospital staff and due to this some women decided to avoid telling the hospital about their decision to homebirth but there were occasions where they found a supportive health professional.

### Preparing for birth

The women in this study prepared for birth in a variety of ways. Some discussed the practical preparation they did in their home for the birth

A few women in the study described how they prepared both physically and mentally for their HBAC by using relaxation techniques and yoga whilst others sought alternative therapies and learnt to manage their own diet and treatments.

The foundation of the preparation was the belief that the woman had an intrinsic ability to birth. Some women expressed how they found this source of birthing knowledge from their own family history, or from other women’s stories and for women that had experience of a vaginal birth before stated that their confidence and knowledge came from understanding how their own bodies had laboured and birthed before.

I’m naturally a confident person, and I knew that I could do it. Especially after having my VBAC in the hospital I thought, god If I could do it at hospital I can do it at home. (Leslie).

The majority of the women had thought about, and mentioned the plans they had, if the need to transfer to hospital arose. Some women identified that the distance from their home to the hospital was a benefit, with the distance ranging from 30 min away to 5 min away.

We took out ambulance cover just in case we wanted to transfer, so we weren’t denying the fact …we prepared for that (Louise).

### Gathering support

As part of the women’s HBAC journey many described four key areas of support, their partner, a doula (or close female friend/relative), a VBAC support group and their private midwife.

All of the women in the study had male partners and enlisting their partner into the decision to homebirth was identified as an important factor. Sometimes women presented the facts to their partner from the available research and sometimes it was the midwife who shared the research evidence with the partners.

I decided I was going to homebirth and my husband was a little bit “oh, I’m not sure” so I told him all the statistics and showed him everything that I had read and the research that its actually safer and these are qualified midwives and I would like to do this process and so he backed me, he said that’s fine if you would like to go down that path I’ll back you (Josephine).

Some women also sought out additional support during labour by engaging a doula (an individual with training in assisting women with natural birth). Reasons given for hiring a doula included the continuity of care that results from the relationship development, as well as having someone with a strong belief in normal birth regardless of the location.

I did hire a doula, which was the best thing I have ever done … I still think if it wasn’t for her I would not have got either my VBAC or my home birth…because she’s just fabulous (Leslie).

Support groups were accessed at a variety of times during the women’s journey to HBAC. Going to a homebirth specific support group allowed for a regular opportunity to have support from other home birthing women. Women described that through a process of becoming immersed in the community and hearing alternative birth stories it allowed for a normalizing of homebirth. The support groups also provided an opportunity for women to meet midwives who supported HBAC. For women that did not have access to face-to-face support groups there was the opportunity to join support groups via social media websites.

### All about safety, but I came first

This subtheme focuses on the woman and her relationship with her private midwife. The majority of the women hired a PPM (10/12). The women in this study who had a PPM found that they accepted their decision-making in a way that did not make the women feel uncomfortable or belittled. Instead of being given prescriptive advice and selected information the women found that the PPM expected the woman to find her own information and draw her own conclusions. Women identified that their PPM gave them more time. The appointments were often much longer than other models of care, ranging from one and a half hours to three hours at a time.

The independent midwives were all about safety but I come first. There’s, no guidelines, there’s no procedures, it’s kind of like tailor made care… even though it was still safety focused, it wasn’t safety slash liability focused …it was about me so that was a big difference (Anne).

During the stresses of labour the PPM is described as remaining calm and supportive as well as flexible and respectful, waiting for the woman to be ready to interact with her and doing that in whatever location and position the woman was in at the time.

My midwife, nine times out of ten, I would not have even remembered she was there, she would just sit back, and observe, you know? (Carol).

### The impact of HBAC

This final theme looks at the sometimes-surprising affects of achieving a HBAC; from the initial euphoria to the way that the women felt they wanted to educate other women. This cohort of women were unique in their varied experience of healthcare providers, models of care and birth location and during the interviews some women found that they reflected on these differences.

### I felt like superwoman

During the semi-structured interviews women were asked the question, “How did you feel after your VBAC at home?” and they unanimously described feelings of empowerment and joy.

Wonderful, so empowered, I could do anything, I knew it was how it was meant to be done, you know, it was just wonderful (Amy).

For some the HBAC brought healing from the previous traumatic birth experience. Some women were surprised by the ease of labour and birth at home and some also felt a sense of pride and achievement. Part of the excitement and accomplishment that women felt involved wanting to announce the birth to the people who had been negative about their decision to HBAC.

You know, it just…it makes me feel so happy, and you know, complete, and, you know, I, I got to enter into this…you know, not, not club but I feel like, you know, this is what I was meant to do, as a woman. (Natalie).

After the HBAC some women found that the way they interacted with their baby and their feelings around motherhood had changed. Some found that they had a deeper bonding experience at home with their baby and that by feeling better after the HBAC they felt better as a mother. There was also an increase in their confidence as a mother. ‘After birthing Jack, who was my second birth at home, I was confident as a mother, I was never confident as a mother with Faith’ (Josephine).

The positive effects of HBAC had a roll-on effect towards the community as the women felt that they had a responsibility to share their experiences and wisdom with other pregnant and birthing women.

I loved making those choices, I loved deciding everything, it’s empowering, that knowledge is empowering. That’s why I want to share our positive homebirth story cos if more women made those choices then the sky’s the limit. (Jeanne).

This was done to avoid other women experiencing the birth trauma that they had and so that other women would know that there is an alternative.

### There is just no comparison

The majority of women interviewed made direct comparisons between their care providers from previous pregnancy and birth and the care provided to them by their PPM. Negative aspects of understaffing and fragmented care contribute to the impersonal nature of mainstream services compared to a PPM who interacted with the whole family unit.

The women were able to articulate the relationship between intrinsic hospital practices and the perceived lack of respect and empathy from the hospital staff.

Women recognized that midwives working within the hospital system placed a greater importance on adhering to hospital policies and procedures rather than focusing on individualised woman-centered care; ‘ I found the midwives to be really lovely, I did find that unfortunately they repeated hospital policy to me more than I needed to hear’ (Louise).

Even when the relationship between the woman and a hospital midwife working in a continuity of care model was good, some women noticed that the hospital policies affected the care given.

The caseload midwife she believed in birth, that was a plus, but she was restricted by hospital policy, so her beliefs didn’t matter. Because she had a job to do and first and foremost was that hospital (Anne).

The fundamental basis of employer loyalties has been suggested as a reason for the different midwifery approaches to care, with women identifying that the PPM worked for the women rather than the health service.

## Discussion

This study has explored women’s reasons for, and experiences of, having a HBAC. The major themes that arose were separated into ‘why it’s never happening again’ and ‘how it’s never happening again’ and these two key points guide the discussion below. The challenge for hospital-based VBAC has been the low success rates of vaginal birth compared to the higher success rates found with HBAC [21]. This study has been able to consider the reasons why women decide to have a HBAC and also explore the factors that might contribute to the success of HBAC. With this knowledge, hospital-based practitioners may be able to gain understanding and compassion for a woman who chooses to have a VBAC which may in turn lead to an increased satisfaction and a better experience for women generally.

### Birth trauma

This study supports previous studies of women’s experience of caesarean section, particularly in relation to birth trauma [12, 13, 37]. Feelings that seem to be attributed to birth trauma include feeling vulnerable, frightened, out of control, ignored and abandoned, anxious, guilt and self blaming [12, 38, 39]. Traumatic feelings about birth have been found to remain with women for many years and are suggested to impact on lifelong self-esteem and willingness to seek healthcare [12, 13, 40]. In the themes, ‘Treated like a piece of meat’ and ‘Traumatised by it for years’, the women reflected on their traumatic birth experience and this became a reason for women to seek a VBAC.

The women did not always choose homebirth as their initial place of birth and many of the women approached hospitals for a VBAC. The findings demonstrated that women were subjected to disrespectful and hurtful attitudes from hospital staff. The negative attitudes of the hospital staff led women to seek alternative options and led them towards, and ultimately to achieve, a HBAC. This is supported by research on women’s experiences of traumatic birth where uncaring attitudes of health professionals were found to be an important influence [13]. Recent WHO guidelines [41] highlight that women commonly experience verbal abuse, coercive medical procedures and a lack of informed consent during pregnancy and birth.

Feminist literature around birth has identified that the abuse and exploitation of women emerges from a patriarchal structured society [42]. This is mirrored in midwifery literature where paternalistic medical practitioners in a medically dominated hierarchical institution practise obstetric care. Women are placed at the bottom of the institutional hierarchy with the expectation that they will agree to the decisions and actions of the presumed experts [42]. Midwifery literature supports the feminist viewpoint that this medicalisation and patriarchy is damaging to women. Midwives working within hospital institutions often find it is more acceptable and easier to conform to the expected medical model than to work against it, thus expecting and encouraging women to conform and capitulate [43, 44]. Women not wishing to conform and to question their position in this hierarchy are then targeted and labelled as ‘difficult’ [45].

### Inflexible guidelines

The women in this study discussed their wish to avoid medical intervention in their births and this contributed to their decision to have a HBAC. This is supported in the literature as a reason for women choosing to homebirth and wishing to avoid interventions such as caesarean section and foetal monitoring [46, 47]. This study supports the notion that one key factor alongside avoiding intervention is a system that is inflexible and hostile to women’s wishes [48, 49].

The focus on managing and monitoring women having a VBAC in hospital is related to the technocratic medicalisation of birth, where increasing advances in technology focuses on the parts of the women being monitored (uterus and baby) with disregard to the wishes and experience of the woman as a whole [42, 50].

### Gathering support

Finding support for their HBAC decision was also an important aspect in preparing for their HBAC. The theme ‘Gathering support’ demonstrates that many of the women found it important to garner the support of their partner, perhaps hire a doula and also find a like-minded group of women via a support group either online and/or face-to-face. Online support groups, social media and blogging are increasingly used forums accessed by women due to their accessibility, commonality and a sense of anonymity [51, 52]. Alongside the gathering of support is the decision to ‘Avoid judgment through selective telling’ regarding their place of birth; this was also found to be a common experience amongst women choosing to homebirth through a publicly funded homebirth program [53].

### Support from their midwife

The women in this study found that the relationship they formed with their private midwife was one based on respect and trust where they were well informed and completely involved with the decision making process. This was represented in the theme ‘All about safety but I came first’. The importance of this respectful relationship is supported by work on redemptive birth [12] with women who have had previous birth trauma [13]. Catling-Paull et al. [53] report that women choosing to homebirth through a publicly funded homebirth program found that the midwives positive approach and respectful attitude contributed to the confidence they had in their midwives and the beneficial relationship that was developed. Women having their first births at home or at hospital also reported that support from midwives was related to the trusting relationship, the time invested in this relationship and the information and communication given by the midwife [54].

The women were able to compare this model of care to the previous types of care they had experienced and found that the continuity of care and the ongoing support were key aspects of this type of care. Continuity of care is well supported in the literature as the gold standard of care not only because women prefer it but also because of the positive effects on normal birth rates, intervention rates and cost [12, 55, 56].

In addition, women identified the difference between having a PPM who was independent of the system, and employed by the woman, compared to one that was employed by the health service. The former was reported as being more supportive and understanding of the woman’s wishes and more likely to encourage the woman to make informed decisions. Exploring this data with a feminist lens it could be suggested that midwives working outside of the hospital system are able to work free from the constraints imposed by oppressive medical practices and policies and were able to reflect the autonomous nature of their work [57]. Alternatively, midwives working within the patriarchal and hierarchal nature of the hospital system can find that it is difficult to act in an autonomous nature and advocate for women [42].

### Study limitations

There are several limitations of this study. The sample was small and comprised a self-selected cohort of women from metropolitan areas of one country. This means that the experiences of these women may not be relevant to women in rural or regional areas, other countries or women who have chosen to have a VBAC in hospital, a birth centre or as part of a public funded homebirth program.

The women in the study were able to access and understand different opinions and statistics around VBAC and this may not be replicated with a different cohort of women with different educational qualifications or access to resources.

Further research is needed to explore this subject more widely. In particular more feminist research is needed to focus on the differences and diversity in women’s voices and acknowledging the role of women’s empowerment through this type of experience [57]. The theoretical principals underpinning feminist research align well with midwifery, including working in partnership with women in an equality-based relationship [42]. Women’s experience of HBAC reported in this paper clearly reflects this partnership approach.

## Conclusion

This research has demonstrated that women often reflect on their previous traumatic birthing experience and seek out alternative birthing options. Restrictive hospital practices and often negative reactions from health practitioners lead women to seek a pathway towards achieving a VBAC and this often leads them to consider a HBAC. Women found that through becoming knowledgeable about VBAC, then finding support and a PPM who would facilitate the HBAC that this inevitably helped them to achieve their HBAC. The HBAC was reportedly both empowering and healing for the women. Exploring this data with a feminist lens revealed that the contemporary paternalistic and medically dominated model of care was often dehumanising and disrespectful compared to the care provided by a PPM where the women described them as behaving in an authentic and autonomous way [42]. A shift in expectation towards encouraging and supporting women who wish to VBAC needs to occur in the health professional community. The voices of women in this study tell us about the importance of relationship and trust in birth and how these can lead to the attainment of a successful VBAC.
